# COVID‐19 pandemic cybersecurity issues

**DOI:** 10.1002/itl2.247

**Published:** 2020-10-14

**Authors:** Bernardi Pranggono, Abdullahi Arabo

**Affiliations:** ^1^ Department of Engineering and Mathematics Sheffield Hallam University Sheffield UK; ^2^ Department of Computer Science and Creative Technologist University of the West of England Bristol UK

**Keywords:** advanced persistent threat, COVID‐19, cybersecurity, internet of things, malware, phishing, ransomware

## Abstract

This paper studies the cybersecurity issues that have occurred during the coronavirus (COVID‐19) pandemic. During the pandemic, cyber criminals and Advanced Persistent Threat (APT) groups have taken advantage of targeting vulnerable people and systems. This paper emphasizes that there is a correlation between the pandemic and the increase in cyber‐attacks targeting sectors that are vulnerable. In addition, the growth in anxiety and fear due to the pandemic is increasing the success rate of cyber‐attacks. We also highlight that healthcare organizations are one of the main victims of cyber‐attacks during the pandemic. The pandemic has also raised the issue of cybersecurity in relation to the new normal of expecting staff to work from home (WFH), the possibility of state‐sponsored attacks, and increases in phishing and ransomware. We have also provided various practical approaches to reduce the risks of cyber‐attacks while WFH including mitigation of security risks related to healthcare. It is crucial that healthcare organizations improve protecting their important data and assets by implementing a comprehensive approach to cybersecurity.

## 1 INTRODUCTION

The COVID‐19 pandemic has created considerable uncertainty, anxiety, and a drastic change as regards our way of life. Organizations have had to adapt to the demand for remote working at speed and scale. Many have been forced to revamp their physical offices and policies which are created in panic to enable employees to work from home without the necessary training or well‐prepared arrangements. Most of these companies and institutions have no plans on the ground to facilitate this drastic and sudden change within a short period.^1^ In fact, only 38% businesses have a cybersecurity policy in place.^1^ By moving to an online environment, organizations and companies worldwide have implemented the work‐from‐home (WFH) business model that increases attack vectors and risks to the internal data. It is worth noting that WFH has become the new normal for people worldwide. In most scenarios, this implies the requirement of employees to use their own personal devices and home networks, which are mostly unsecured by nature and lack the required industrial standard security measures. For institutions that already provide their employees with business devices, these are typically secured with minimal or no administrative rights. Conversely, the general setup where staff are given temporary rights to install the required software becomes an issue. Hence, businesses need to provide more realistic solutions and provide employees with more rights, which indirectly implies more potential security issues.

Cybersecurity during the coronavirus disease 2019 (COVID‐19) pandemic is a genuinely concerning issue on account of the emerging cyber‐threats and security incidents targeting vulnerable people and systems globally.^2^ This paper focuses on the cybersecurity issues that have emerged in various environments in the wake of the global pandemic.

## 2 BACKGROUND

Even under normal circumstances, online crimes such as scams provide better returns with the least risk for the attackers. Examining the fact, more people are now unemployed, spend more time at home and use the Internet for work and to socialize. Furthermore, governments have provided incentives to help people financially and so also other business to seek to attract or retain customers. As the world anticipates a potential cure to control the spread of COVID‐19, all information related to “COVID‐19” will gain the attention of netizens. The scammers are taking advantage of this avenue to send malicious [phi, smi, vi] shing^3^ attacks to victims disguised as the government, tax authorities, etc. with links to claim assistance in relation to COVID‐19.

In its report, the World Economic Forum (WEF) highlighted that hacking and phishing is the new norm. Even after the viruses have disappeared.^4^ These scams are much more effective now during the pandemic as most vulnerable people are more anxious and expecting emails, text, calls, etc. relating to COVID‐19 from the authorities. As cyber criminals become more aware of this situation, it is much easier for them to create fake messages or websites that replicate the appearance of relevant and familiar authorities, incorporating words that use urgency to exploit the globally felt fear factor due to the importance of handling an emergency and needs. Therefore, cyber criminals can increase the effectiveness of their phishing attacks. These attacks can come in various forms, such as internal and external updates, personal gains, and charity. A recent study from F‐Secure highlighted that spam is one of the common ways to spread malware. It also pointed out how attackers are using the pandemic to entice people to click, primarily by hiding the executable in archive files such a .zip files.^5^ It should be mentioned that malicious actors may use existing, genuine materials as bait to encourage people to perform a risky action such as click on a link or open an attachment. It is essential that users look at the sender of an email and examine any links contained within it prior to acting. Cyber criminals often use impersonation techniques posing as the World Health Organization (WHO), United Nations (UN) or a popular company whilst people are WFH, Zoom, to trick users into clicking on links or to open infected documents.

As a result of the pandemic, we have seen a total lockdown in almost all parts of the world. The shift to the new way of working where employees are working from home primarily using their home systems which are secured by their employers has created a degree of concern within the sector. Owing to this mass quarantine arrangement, new challenges pertaining to the resilience of technological solutions to most ecosystems is vital; specifically, the resilience of current technology within employers' existing cyber infrastructures.

## 3 CYBERSECURITY ISSUES DURING THE COVID‐19 PANDEMIC

### 3.1 Cyber‐attacks during the COVID‐19 pandemic

Cyber‐attacks during the pandemic can be categorized into three categories: scams and phishing, malware, and distributed denial‐of‐service (DDoS). Certain examples of cyber‐attacks during the pandemic are outlined in Table 1. Cyber criminals and Advanced Persistent Threat (APT)^6^, ^7^ groups are launching cyber‐attacks at vulnerable people and organizations via COVID‐19 related scams and phishing. They are exploiting the pandemic for various motivations, for instance for commercial gain or to collect information related to COVID‐19 vaccines by deploying different techniques such as phishing or ransomware and other malware. Examples of APT activities during the pandemic include Hades, Patchwork (aka Dropping Elephant, APT‐C‐09), TA505,^8^ and APT29.^9^

- Scams and Phishing: The most common and effective attack during this pandemic is via different types of scams and phishing.^10^, ^11^ In fact, phishing attacks have a success rate of 30% or higher. It is extremely troubling that an attacker only requires a small percentage of clicks to make financial gains or other interests. Therefore, sending millions of emails to victims who are seeking to apply for funding relief provided by the government, their employers, banks, etc., will result in swift and enormous rewards. There are various phishing attacks (email, SMS, voice) targeting vulnerable people and systems using coronavirus or COVID‐19 as a title to entice people.^10^, ^11^ There were an increase of 600% coronavirus‐related phishing email attacks in Q1 2020.^12^ Cybercriminals also use more sophisticated techniques to lure victims such as using HTTPS encryption protocols in their websites. In fact, around 75% of phishing sites have been equipped with SSL.^11^ Additionally, webmail and Software‐as‐a‐Service (SaaS) users are the most‐targeted phishing sectors.^11^
- Malware: Malware includes computer viruses, worms, a Trojan horse, spyware, and ransomware.^13^ During the pandemic, cyber criminals and APT groups have taken advantage in targeting vulnerable people and systems by spreading various types of malware through emails and websites. In fact, 94% of computers corrupted by malware were infected by an email. Specific types of malware,^14^ such as ransomware will be more effective for institutions that are heavily involved in dealing with the pandemic (see Table 1).
- Distributed Denial‐of‐Service (DDoS): Due to its simplicity to launch attacks and its impact on the victim, a DDoS attack is considered as the most indefensible cyber‐attack today. Unlike traditional denial‐of‐service (DoS) attacks, a DDoS attack exploits numerous attack sources, is spread using multiple hosts to launch a coordinated DoS attack against one or more targets which effectively intensifies the attack power and makes defense more complicated.^15^ In the UK, universities' Internet service provider JISC experienced a DDoS attack during the pandemic, disrupting students and staff access to university IT resources and the Internet. Moreover, it is important to note that DDoS attacks are also being exploited to undermine health organizations worldwide (see Table 1).

**TABLE 1:** Examples of cyber‐attacks during COVID‐19 pandemic

Date	Country	Type of attack	Details of attack
March 2020	Czech Republic	Ransomware	The Brno University Hospital as one of COVID‐19 testing laboratories in the country has been hit by a cyber‐attack and was forced to shutdown its entire IT network.^16^
March 2020	UK	Ransomware	The Maze ransomware group has published personal and medical details of thousands of former patients of a London‐based medical research company which provide COVID‐19 testing.^17^
March 2020	France	DDoS	The systems of a group of hospitals in Paris which plays an important role in fighting COVID‐19 crisis in the capital were the target of a DDoS attacks disrupted access to server and email.^18^
March 2020	US	DDoS	The US Department of Health and Human Services Department which heavily dealing with COVID‐19 issue in the country were the target of a DDoS attacks.^18^
May 2020	Taiwan	Phishing	Emails contained a remote access hacking tool impersonating Taiwan's top infection‐disease official urging recipients to get coronavirus tests.^19^
June 2020	Germany	Phishing	Phishing emails to senior executives at the company which supply personal protective equipment (PPE). The phishing links were designed to direct executives to fake Microsoft login pages to steal their credentials.^20^
June 2020	US	Ransomware	The University of California San Francisco (UCSF) which working on COVID‐19 vaccine was the target of a ransomware attacks and forced to pay $1.14 m to cybercriminals called Netwalker.^21^
June 2020	Canada	Ransomware	CryCryptor ransomware masquerades as COVID‐19 contact‐tracing apps on Android device.^22^

### 3.2 Cyber‐attacks on healthcare organizations

The healthcare sector has been one of the main targets of cyber‐attacks during the pandemic. The hacking attempts on healthcare organizations has highlighted the problems associated with cybersecurity in the healthcare sector. These include healthcare bodies, pharmaceutical companies and research organizations. Healthcare organizations are vulnerable to cyber‐attacks, such as the WannaCry ransomware attack that incapacitated the National Health Service (NHS) in 2017. One of the main reasons is due to limited budgets these organizations have to protect their IT systems as they are funded by cities or countries which typically under very strict budget controls. For example, many healthcare organizations still operate outdated software or no longer supported operating system (OS) like Windows 7 or Windows XP to control medical devices throughout the hospitals. In fact, Europol stated that healthcare facilities are considered an easy and profitable target for ransomware. Nowadays, modern hospitals are run by computers. Computers and the Internet of Things (IoT) are utilized heavily in modern hospitals to store and monitor patients' data as well as to control medical devices such as an intensive care unit (ICU) or ventilators.

A joint advisory report and guidelines from the United Kingdom's National Cyber Security Centre (NCSC) and the United States Department of Homeland Security (DHS) Cyber Security and Infrastructure Security Agency (CISA) provided discussion on issues such as phishing, malware, the tools used in WFH such as Zoom, etc.^10^ It is predicted that APT groups will continue to target healthcare and essential services globally.^23^ A recent joint advisory report from NCSC and Canada's Communications Security Establishment (CSE) strongly suggested that the Russian intelligence services are behind the APT29 (aka “Cozy Bear”) cyber‐attacks on various organizations dealing with the development of a COVID‐19 vaccine in Canada, the US, and the UK, with the aim of stealing COVID‐19 vaccines' related information.^9^ To achieve its goals, APT29 uses various techniques, such as vulnerability scanning, public exploits and phishing to gain access to the target network and custom malware known as ‘WellMess’ and ‘WellMail’ to carry out further damage.^9^

### 3.3 Mitigation

Mitigating and preventing cyber‐attacks are not a trivial task. There are practical approaches that can reduce the risk of cyber‐attacks while WFH^1^, ^10^, ^23^:

- User Education: Security is only as strong as its weakest link. People are considered the weakest link in many security systems. Therefore, developing cybersecurity awareness among users by means of constant training is important to reduce the risks of cyber‐attacks on an organization. A recent study shows that only 11% businesses have provided cybersecurity training to non‐cybersecurity employees in the past year.^24^
- Virtual Private Network (VPN): VPN is an encrypted communication channel between two points on the Internet to protect the data that is sent and received. The use of a VPN to surf the Internet is the new normal. A VPN provides two aspects of security: confidentiality and integrity and allows organizations to extend security policies to remote workers.
- Enable multi‐factor authentication (MFA): MFA strengthens security by requiring a username and password plus a one‐time code sent to mobile phone via SMS or an authentication app. MFA is an important factor to mitigate against password guessing and theft such as brute force cyber‐attacks. An employee attempting to access her company's network from home will need to provide both her username and password and a one‐time code sent to her mobile phone to verify her identity before being allowed to access the internal network.
- Ensure all devices firmware is up‐to‐date: Ensure that all devices and equipment firmware/OS are up‐to‐date with the latest security patches implemented to inoculate them against known vulnerabilities. Regular and up‐to‐date patches may reduce the risk of a zero‐day attack.
- Ensure that up‐to‐date anti‐malware software is activated in all network connected devices: Cyber criminals targeting vulnerable people by spreading various types of malware. As millions of new malware and its strain are generated every year, regular and up‐to‐date anti‐malware may reduce the risk of cyber‐attacks caused by malware.
- Enable strong company online policy: Organizations have had little or no time to prepare for the WFH scenario. Robust and comprehensive WFH policy is necessary to protect data and prevent cyber‐attacks. Strong WFH policies include avoiding holding sensitive work conversations in public, use only company‐approved video and audio conference lines, etc. The policies should also include a robust and proven recovery plan and backup strategy. It is also essential to have these plans a regular test as a recent study highlighted that 46% businesses only test their recovery and backup plans once a year or less.^25^
- Segmentation and separation: Move away from an “all‐in‐one” single purpose device and network. Divide a network into different trusted zones: home office network (high trust level), guest and home entertainment network (low trust level) and Internet zone (untrusted). In smart homes, the IoT devices should be isolated in a separate Wi‐Fi network. By isolating the IoT devices on a separate network segment, any compromise of an IoT device will not automatically grant access to a user's primary devices such as a corporate laptop.
- Physical security of home office: It is important to physically protect home office devices. Practical approaches include ensuring that work devices are not left unattended, use a lock screen or lock the laptop, always log off devices after use, etc.

In addition to the general mitigation approaches discussed above, an example of the mitigation of security risks related to healthcare is outlined below. During the pandemic, healthcare organizations dealing with COVID‐19 have been the principal target of persistent cyber‐attacks. It is imperative that healthcare organizations protect their valuable data and assets from cyber‐attacks by improving their defense. Two important components as regards detecting malicious behavior that can compromise the security and trust of a network are intrusion detection system (IDS) and security incident and event management (SIEM). Typically, an IDS employs anomaly detection, stateful protocol analysis (aka deep packet inspection), signature matching or a combination of all three techniques (hybrid) to analyze incoming cyber‐attacks. Due to its ability to detect zero‐day attacks more accurately, AI‐based anomaly detection IDS is growing in popularity to detect cyber‐attacks. Furthermore, it is important for healthcare organizations to take a comprehensive approach to cybersecurity and not to view security from a technological perspective only, but in the framework of processes.^26^ Examples of a comprehensive approach to cybersecurity include the CERT Resilience Management Model (CERT‐RMM),^27^ risk management, and incorporating cybersecurity into the strategic planning and budgeting process.^26^

## 4 CONCLUSION

In this paper, cybersecurity issues during the COVID‐19 pandemic have been discussed and analyzed. Notable cyber‐attacks and vulnerabilities are highlighted and summarized. Certain practical approaches to reduce the risks of cyber‐attacks and possible mitigation techniques are also discussed.

During this pandemic, cyber criminals and APT groups have taken advantage of targeting vulnerable people and systems. Furthermore, it is a situation that is unlikely to change in the foreseeable future. Healthcare organizations are one the main victims of cyber‐attacks during the pandemic for various reasons. Hence, it is crucial that healthcare organizations improve protecting their important data and assets from cyber‐attacks by leveraging their defense such as implement comprehensive approach to cybersecurity.
